# Understanding incomprehensibility: Misgivings and potentials of the phenomenological psychopathology of schizophrenia

**DOI:** 10.3389/fpsyg.2023.1155838

**Published:** 2023-03-28

**Authors:** Hannes Wendler, Thomas Fuchs

**Affiliations:** ^1^Department of Philosophy, University of Cologne, Cologne, Germany; ^2^Phenomenological Psychopathology and Psychiatry, University Clinic Heidelberg, Heidelberg, Germany

**Keywords:** schizophrenia, obscurity, inexplicability, Karl Jaspers, Ludwig Binswanger, phenomenological psychopathology, praecox feeling, un-understandability

## 1. Introduction

Incomprehensibility is canonically regarded a key characteristic of schizophrenia. Bizarre delusions, in particular, contribute to its clinical picture and have been considered essential for diagnosing schizophrenia. Accordingly, the DSM-IV-TR speaks of bizarre delusions “if they are clearly implausible and not understandable and do not derive from ordinary life experiences” (American Psychiatric Association, 2007, p. 299). The ICD-10, on the other hand, complements that bizarre delusions are “culturally inappropriate and completely impossible” (World Health Organization, 1992, p. 87). In light of this, schizophrenia makes for the paradigm case of a *psychopathological shift in consciousness*, which has been described in terms of “*a transformation in our total awareness of reality*” (Jaspers, 1997, p. 95) or an “altered framework for experiencing” (Parnas and Henriksen, 2013, p. 320). The enigmatic character of this psychopathological shift consists in its all-encompassing nature, boiling down to its “core Gestalt” of “a fundamentally changed subjectivity that may manifest itself across all mental domains: affect, expression, motivation, mood, cognition, willing and action” (Parnas, 2012, p. 68). Since this shift consists in a pronounced instability of the schizophrenic *self* (Henriksen et al., 2021; Burgin et al., 2022), it is subject to debate whether it is best conceived as an *explorable transformation of consciousness* or as its *unfathomable disorganization*. This question has troubled the psychopathological discourse on schizophrenia significantly (Andreasen and Flaum, 1991; Parnas, 2011; Henriksen, 2018), in spite of the widespread recognition of the clinical utility of the notions of incomprehensibility and bizarreness (Cermolacce et al., 2010; Feyaerts et al., 2021).

The conundrum of schizophrenic incomprehensibility consists in whether there is any meaningful sense in which we can *understand this incomprehensibility*. We believe that there is. However, accessing the phenomenon of schizophrenic incomprehensibility is hindered by several confusions surrounding the psychopathological discourse.

In order to arrive at an unclouded judgement, the confusion surrounding the issue of incomprehensibility must itself be investigated. We propose that this confusion stems from three distinct sources. In the following we elaborate on each of them and advance a scheme for structuring the discourse on schizophrenic incomprehensibility (see Table 1):

1. *Overreliance on delusional beliefs*. The problem of incomprehensibility is ill-posed, biasing the discourse toward the *delusional beliefs* as is evidenced by their characterization in the ICD and DSM. Consequently, the *origin and the experience of delusions* are overlooked. Since they lie at the root of the psychopathology of schizophrenia, the discourse on the origin and experiential structure of incomprehensibility must be revisited.
2. *False threat of irrationalism*. Acknowledging the clinical reality of schizophrenic incomprehensibility is misevaluated as endangering the scientific status of psychopathology by pushing it toward irrationalism. Such an evaluation ultimately hinders the project of determining the possibilities and limits of psychopathological knowledge, which is essential to establishing it as a strict science: In light of the phenomenological approach, schizophrenic incomprehensibility does not mark the endpoint of our understanding of schizophrenia but is a starting point for developing a *psychopathological agnotology* (i.e., the scientific investigation of the production and experience of incomprehensibility).
3. *Equivocations*. The discourse on incomprehensibility is riddled with equivocations. This means that conflating concepts such as un-understandability, oddity, schizophrenic alterity or the praecox feeling is the norm rather than the exception. In order to distinguish these related concepts, it is helpful to consider their intellectual origins and to systematically classify competing approaches to schizophrenic incomprehensibility. Considering incomprehensibility can aid in enriching the discourse by moving beyond the classical framing in terms of the understanding-explanation dichotomy to the more adequate and encompassing trichotomy of *un-understandability* (“*Unverständlichkeit*”), *obscurity* (“*Uneinfühlbarkeit*”) and *inexplicability* (“*Unerklärbarkeit*”).

**Table 1:** A scheme for structuring the debate on schizophrenic incomprehensibility.

**Theory**	**Concept**	**Obscure**	**Un-understandable**	**Inexplicable**	**Psychopathological shift in consciousness**
General psychopathology	Primary phenomenon (“*Urphänomen*”)	Yes	Yes	Yes (experience) Preliminary (origin)	Disorganization
*Daseinsanalyse*	Mode of being-in-the-world	No	No	Preliminarily	Transformation
Phenomenological psychiatry	Ipseity-disturbance model	Yes	No	Preliminarily	Transformation

The debate on schizophrenic incomprehensibility is troubled by several equivocations. The scheme above proposes a remedy by distinguishing whether incomprehensibility refers to an impossibility to empathize with schizophrenic patients (obscurity, “*Uneinfühlbarkeit*”), an impossibility to understand schizophrenic experiences (un-understandability, “*Unverständlichkeit*”) or an impossibility to explain schizophrenia (inexplicability, “*Unerklärbarkeit*”). This framing can be applied to historical and contemporary approaches to schizophrenic incomprehensibility and helps to systematize the discourse on psychopathological shifts in consciousness.

In what follows, we sketch how phenomenology can aid psychopathology in overcoming these idols and, ultimately, arrive at a more encompassing and adequate assessment of schizophrenia. This entails that not only the clinical reality of schizophrenic incomprehensibility must be acknowledged, but—beyond that—investigating its experiential structure (both, of the patient and the clinician) is of the essence.

## 2. Overreliance on delusional beliefs

In order to outline a potential remedy for the bias toward delusional beliefs, we first turn to a historical perspective. Spitzer et al. (1993) notes that the concept of bizarre delusions derives from Kraepelin characterizing schizophrenic delusions as “non-sensical” and from Jaspers deeming them “incomprehensible” (cf. Cermolacce et al., 2010). The latter also originated the *standard view* of schizophrenic delusions, according to which they are conceived of as false beliefs that cannot be corrected and are entertained with subjective certainty (Jaspers, 1913a; Parnas, 2012). This standard view was maintained in the ICD's and DSM's insistence on the impossible contents of delusional beliefs until recently (cf. Heinimaa, 2002). Consider, for instance, the DSM-IV-TR's definition of schizophrenic delusions: “Delusions are erroneous beliefs that usually involve misinterpretation of perceptions or experiences” (American Psychiatric Association, 2007, p. 275–276). Whereas contemporary treatments focus on the incomprehensibility of the delusional *content*, i.e., the falsity, robustness and certainty of the propositional belief, Jaspers, originally, was concerned more with the *origin* and the *experience of delusions* (Jaspers, 1913a,b; cf. Schmitt, 2018).

With regard to this, three different notions of incomprehensibility ought to be differentiated. The first one derives from Jaspers' interpretation of Dilthey (1894) methodological dualism (cf. Henriksen, 2013). Since Jaspers posits a somatic origin of delusions, their scientific investigation ought to treat them as causal-genetic objects of explanation (cause-effect; nexus of causality). Accordingly, incomprehensibility pertaining to the origin of delusions arises because of the *categorical inapplicability of understanding*, which presupposes a meaningful psychological motivation through previous experiences (purpose-consequence; nexus of finality). Thus, a failure to identify the somatic origins of schizophrenia is more aptly described in terms of *inexplicability* (“*Unerklärbarkeit*”), which depends on the progress of the natural sciences and, accordingly, might be merely temporary.

The second and third notion of incomprehensibility both pertain to the status of schizophrenic delusions as primary phenomena (“*Urphänomene*”) (cf. Heinze and Kupke, 2006; Kupke, 2008; Thoma, 2013). One the one hand, primary delusions that occur in schizophrenia amount to an immediate, perception-like “awareness of meaning” that “undergoes a radical transformation” (Jaspers, 1997, p. 99). This entails that such primary delusions, in contrast to delusion-like ideas, cannot meaningfully be traced back to ‘the content' of preceding mental states and, thus, are ‘unmotivated' or exhibit no ‘meaningful connections'. Therefore, primary delusions are *un-understandable* in the sense that they *defy the purpose-consequence structure* of the nexus of finality.

On the other hand, primary delusions encompass changes on the level of subjectivity (Owen et al., 2004). Such primary delusions are disorders of self-consciousness and object-consciousness, such as thought broadcasting, thought insertion, delusions of passivity, etc. Since they pertain to the sphere of the conscious experience of reality (“*Wirklichkeitserleben*”), they cannot be reduced to or analogized with other phenomena but unveil a primary stratum of existence. For this very reason, Jaspers holds that primary delusions lie outside the realm of science altogether and must, instead, be investigated philosophically (Kupke, 2008). Hence, incomprehensibility concerning delusional experience arises because primary delusions exceed the scope of scientific investigation and, accordingly, *lie beyond the dichotomy of explanation and understanding*.

Considering this third sense, incomprehensible delusional content (as well as “crazy actions”), in turn, would be conceived of as a manifestation of the underlying primary delusional experience and has only a secondary status. This implies that it would be a mistake to take impossible delusional content as a sufficient criterion for diagnosing schizophrenia. Instead, “we must realize that the content and structure of these experiences are dialectically intertwined, and therefore we must take into account the altered framework of experiencing in schizophrenia” (Parnas and Henriksen, 2013, p. 324).

## 3. False threat of irrationalism

Explicating the changes of the experiential structure in schizophrenia converges with the prime interest of its phenomenological treatment. In the recent discourse, researchers agree that the psychopathological shift in consciousness occurring in schizophrenia can be described as a disturbance on the level of the *minimal self* (Cermolacce et al., 2007; Hur et al., 2014; Nelson et al., 2014), i.e., an abnormal sense of the first-person quality of experience, a loss of “mineness” that can lead to a quasi-solipsistic world-view and a pervasive alienation from the lived-body, i.e. disembodiment (Fuchs, 2020b). This disordered structure underpins changes (a) on the level of the *extended self* (Gallagher, 2003; Phillips, 2003; Parnas and Zandersen, 2018), i.e., a fragmented or delusional narrative self-understanding that becomes explicit in schizophrenic belief contents, and (b) on the level of *extended intersubjectivity* (Stanghellini and Lysaker, 2007; Fuchs, 2010; Frith, 2015; Gallagher and Varga, 2015; Van Duppen, 2017), i.e., difficulties in participating in conversational exchanges, explicit other-understanding *via* theory of mind, and an intense sense of threat coming from the social realm. In sum, the outlook of phenomenological psychopathology can help reorient the discourse on schizophrenia from its surface level features (delusional belief content) back to the underlying changes in the structure of experience.

What can such a phenomenological outlook contribute to understanding incomprehensibility in schizophrenia? First of all, conceiving of schizophrenia as an altered framework for experiencing allows to identify “a developmental continuity from early non-psychotic self-disorders to the fully formed first-rank symptoms” (Parnas and Henriksen, 2013, p. 324). It is important to note that this continuity is neither one of physical causation (nexus of causality), nor one of mental motivation (nexus of finality), but rather an *eidetic continuity* (Parnas and Henriksen, 2013). Hence, the ipseity disturbance model (Nelson et al., 2014; Nordgaard et al., 2023) conceives of schizophrenia in terms of a disorder at the level of the minimal self and attempts to identify experiential structures that are present in the sub-clinical and clinical picture of the disorder. In terms of a phenomenological act-analysis, this means that the disturbance on the level of the minimal self-corresponds to a dialectical process in which perturbations of the intentional structure of experience (e.g., an excessively self-referential act-structure) elicit compensatory symptoms (e.g., hyperreflexivity or excessive introspection) and disturbances of the pre-reflective, passive synthesis of meaning.

Delusional belief contents, then, can be viewed as an attempt to thematize these underlying changes and, hence, exhibit a so-called “delusional logic” (“*Wahnsinnslogik*”) (Wulff, 1992). By unearthing these foundational layers to psychopathological shifts in consciousness, phenomenological psychopathology contributes not only to a better understanding of the patient's experience from his or her own perspective, but also offers conceptual and methodological means for the early detection of schizophrenic psychosis (Parnas et al., 2005; Sass et al., 2017), which is sometimes prematurely reserved for neurobiological approaches to psychopathology (Insel, 2010; Heinssen and Insel, 2015). By shedding light on this eidetic continuity, phenomenological psychopathology provides a framework that furthers scientific understanding of incomprehensibility by illuminating its development.

Over the course of the discourse's development, the “theorem of incomprehensibility” (Kupke, 2008)—sometimes also referred to as Jaspers's theorem—has been criticized and ultimately rejected by several competing psychopathological approaches, for instance, systems approach (Bateson et al., 1956) and psychoanalysis (Freud, 1911), but also other, phenomenologically inclined approaches such as anthropological psychiatry (Zutt, 1963) or *Daseinsanalyse* (Binswanger, 1957). The very concept of incomprehensibility has been perceived to push psychopathology toward irrationalism and, correspondingly, acknowledging schizophrenic incomprehensibility has been equated to abandoning the scientific enterprise altogether. Before this backdrop, the concept of incomprehensibility was reduced to that of delusional content and psychopathological interest in the notion has shrunk down to its operational value for diagnosis.

Why, then, did Jaspers and his successors insist on maintaining the concept of incomprehensibility in phenomenological psychopathology?

Firstly, an overemphasis on resolving incomprehensibility runs the danger of misconstruing the clinical picture of schizophrenia. Schizophrenic incomprehensibility lies at the root of nothing less than what Jaspers holds to be “[t]he most profound distinction in psychic life,” namely “that between what is meaningful and allows *empathy* and what in its particular way is *ununderstandable*, ‘mad' in the literal sense, schizophrenic psychic life” (Jaspers, 1997, p. 577). Accordingly, this pertains to “the basic problem of psychopathology” (Jaspers, 1997, p. 702) that consists in learning to differentiate unified personality developments from disruptive processes that break with life's continuity.

“[T]he facts are overlooked in an endeavor to see the individual as understandable […]. [W]e […] should recognize what is not understandable in all its complex heterogeneity and grasp it methodically according to what its nature may be” (Jaspers, 1997, p. 705).

Therefore, recognizing schizophrenic incomprehensibility must not be confused with giving up on its psychopathological investigation, but rather is an integral part of a strict and sober *clinical description*.

This brings us, secondly, to the potential of establishing a *psychopathological agnotology* that revolves around the concept of incomprehensibility. This means that acknowledging incomprehensibility is no longer viewed as an endpoint of the scientific treatment of schizophrenia, but as marking the starting point of a new field that differentiates forms of incomprehensibility and investigates the mechanisms that underlie and produce it. Not unlike the adventurers of the Age of Discovery, who charted unknown parts of the globe, the so-called *terra incognita*, psychopathological agnotology can provide guidance and direction for studying schizophrenic incomprehensibility, analogously: *mens incognita*. In its strongest form, however, such a psychopathological agnotology goes beyond the mere mapping of what might one day be rendered understandable and homes in on the “positive message of incomprehensibility” (Wulff, 1992, p. 7; cf. Schlegel, 1800; Bauer, 2011).

For the most part, this remains a desideratum for further research. Nevertheless, genetic phenomenology and the analysis of disturbed patterns of passive synthesis in schizophrenia afford promising research perspectives. In this vein, Wulff spells out the delusional logic in terms of “acts of paradoxicalization” (1992, p. 9) that describe how subjective-situational meaning (“*Sinn*”) and objective-general meaning (“*Bedeutung*”) become decoupled and reconfigured. Similarly, Moskalewicz and Gozé turn to a genetic analysis of “bizarreness of contact” (Moskalewicz and Gozé, 2022, p. 144) as a pre-reflective and ante-predicative atmospheric quality that surrounds the encounter with schizophrenic patients and corresponds to Rümke's (1941) infamous praecox feeling by the clinician (cf. Varga, 2013; Gozé and Naudin, 2017). Relatedly, Fuchs (2020a) advanced a genetic analysis from an enactive perspective that conceives of the experiential change at the beginning of psychosis in terms of a subjectivization of perception that results in a disembodiment and derealization of experience. Instead of capitulating before schizophrenic incomprehensibility, genetic phenomenology provides the theoretical scaffolding for acknowledging and analyzing its experience.

## 4. Equivocations

Considering the phenomenological discussion of the praecox feeling is telling, because it allows to shed light on an equivocation troubling schizophrenia research (see Table 1). The praecox feeling has been described as a feeling of bizarreness and unease when encountering schizophrenic patients and, ultimately, as the impossibility of empathizing with them (Rümke, 1941). Albeit being subject to considerable criticism, both concerning the prospect of its phenomenological rehabilitation (Parnas, 2011) and regarding its empirical and diagnostic validity (Grube, 2006; Gozé et al., 2019), the notion of the praecox feeling has recently been reconsidered in light of interactionist interpretations of direct perception theory of empathy (Haker and Rössler, 2009; Gallagher and Varga, 2015). Within this framework, the praecox feeling is explicated as a lack of interaffective and interbodily resonance, ultimately leading to a breakdown of enactive sense-making and social understanding (Varga, 2013). Hence, the patient's schizophrenic disembodiment is *empathically experienced* by the clinician *through* the praecox feeling (Fuchs, 2020b) or, following Moskalewicz and Gozé (2022), the preceding “bizarreness of contact.”

From a historical perspective, the debate concerning the praecox feeling connects to the discourse on *obscurity* (“*Uneinfühlbarkeit*”), viz. the *impossibility to empathize*. In the beginning of the 20th century, a controversy ensued regarding the conceptualization of schizophrenic incomprehensibility within phenomenological psychopathology (Schmitt, 2018), sometimes referred to as the *Jaspers-Binswanger controversy* (Basso, 2016). Essentially, Binswanger (1913; 1914, cf. 1957) opposed Jaspers' theorem of incomprehensibility and conceived of schizophrenia as a specific and deficient, yet understandable mode of being-in-the-world. Binswanger's (1913, 1914) and Jaspers (1913a,b) exchange during 1913–1914 was embedded in ongoing debates in the vicinity of Kraepelin's and Bleuler's schools as well as the broader context of the method dispute that started at the end of the 19th century. A number of psychopathologists influenced by Scheler's notion of sympathy, Bergson's concept of intuition and Heideggers' term of being-with took issue with Jaspers' framing of schizophrenic incomprehensibility *via* the distinction between static and genetic understanding, since it remained indebted to Dilthey's understanding, Lipps's *Einfühlung* and Freud's interpretation (cf. Kupke, 2008; Henriksen, 2013).

Indeed Jaspers' notion of incomprehensibility never properly connected with the phenomenological tradition of empathic other-experience that originated with Scheler's (1913) proposal of unmediated expression-perception, i.e., in the same year as Jaspers' psychopathology was first published. Accordingly, Minkowski (1927) proposed a “diagnosis by penetration,” Wyrsch (1946) advanced the notion of “diagnosis by intuition” and Binswanger (1955) argued for a “diagnosis by feeling” (cf. Parnas, 2011; Moskalewicz and Gozé, 2022):

“The question of whether the psychic life of the mentally ill follows the same laws as that of healthy people is intimately connected to the question of whether and to what extend we can empathize [“einfühlen”] with the psychic life of the mentally ill; with other words: we will approximate a decision regarding this question to the degree that we learn to empathize with the psychic life of the mentally ill” (Binswanger, 1914, p. 596).

On the one hand, these concepts aimed at establishing a specific role of empathy in conceiving of schizophrenic incomprehensibility that is distinct from the role of understanding. On the other hand, they challenged Jaspers's theorem of radical incomprehensibility and attributed a broader epistemic scope to the phenomenological analysis of schizophrenia. Taking this into account and connecting it with the argument from (1), the canonical framing of the Jaspers-Binswanger controversy as evolving around the understanding-explanation dichotomy can be recast in terms of a trichotomy of empathy-understanding-explanation. Consequently, this allows to differentiate three senses of schizophrenic incomprehensibility that are routinely conflated, namely obscurity (“*Uneinfühlbarkeit*”), un-understandablility (“*Unverständlichkeit*”) and inexplicability (“*Unerklärbarkeit*”; see Table 1). Learning to structure the debate accordingly is helpful for accounting for whether schizophrenic shifts in consciousness are best conceived of as explorable transformations or unfathomable disorganizations.

## Author contributions

All authors listed have made a substantial, direct, and intellectual contribution to the work and approved it for publication.
